# Complement Inhibition Therapy in Myasthenic Crisis—A Multicentre Retrospective Analysis of 17 Cases From Germany

**DOI:** 10.1111/ene.70596

**Published:** 2026-04-15

**Authors:** Lea Gerischer, Maike Stein, Alice Schneider, Hanna Tangen, Johanna Loris, Stefanie Glaubitz, Jana Zschüntzsch, Ulrich Hofstadt‐van Oy, Charlotte Schubert, Christoph Heesen, Melina Schlag, Tim Hagenacker, Menekse Oeztuerk, Tobias Ruck, Paolo Doksani, Carla Dusemund, Meret Herdick, Julia Herzig‐Nichtweiß, Philipp Mergenthaler, Frauke Stascheit, Amani Suboh, Lisa Schwarz, Sarah Hoffmann, Andreas Meisel, Sophie Lehnerer

**Affiliations:** ^1^ Department of Neurology With Experimental Neurology Charité – Universitätsmedizin Berlin, Corporate Member of Freie Universität Berlin and Humboldt‐Universität zu Berlin Berlin Germany; ^2^ Neuroscience Clinical Research Center Charité – Universitätsmedizin Berlin, Corporate Member of Freie Universität Berlin and Humboldt‐Universität zu Berlin Berlin Germany; ^3^ Berlin Institute of Health at Charité – Universitätsmedizin Berlin Digital Health Center Berlin Germany; ^4^ Institute of Biometry and Clinical Epidemiology Charité – Universitätsmedizin Berlin, Corporate Member of Freie Universität Berlin and Humboldt‐Universität zu Berlin Berlin Germany; ^5^ Department of Neurology University Medical Center Göttingen Göttingen Germany; ^6^ Department of Neurology Knappschaft Kliniken Dortmund Dortmund Germany; ^7^ Institute of Neuroimmunology and MS (INIMS) and Department of Neurology, University Medical Center Hamburg‐Eppendorf Hamburg Germany; ^8^ Department of Neurology and Center for Translational Neuro‐ and Behavioral Sciences (C‐30 TBNS), university Medicine Essen Essen Germany; ^9^ Department of Neurology, Medical Faculty and University Hospital Düsseldorf Heinrich Heine University Düsseldorf Düsseldorf Germany; ^10^ Department of Neurology With Heimer Institute for Muscle Research University Hospital Bergmannsheil Bochum Germany; ^11^ Center for Stroke Research Berlin Charité – Universitätsmedizin Berlin Berlin Germany; ^12^ Radcliffe Department of Medicine University of Oxford Oxford UK

**Keywords:** C5‐inhibition, eculizumab, myasthenic crisis, neurocritical care, ravulizumab

## Abstract

**Introduction:**

Generalized myasthenia gravis (gMG) may progress to life‐threatening myasthenic crises (MC) requiring mechanical ventilation. Standard therapy includes intravenous immunoglobulins (IVIg), plasmapheresis (PLEX), immunoadsorption (IA), and high‐dose corticosteroids. This study aimed to evaluate the real‐world effectiveness of complement‐inhibitors (C5‐I) in patients refractory to the standard treatment of MC (termed therapy‐refractory MC).

**Methods:**

This multicentre, retrospective study included patients with acetylcholine‐receptor‐antibody positive (AChR‐ab+) gMG experiencing MC or severe exacerbations (MGFA IVb/V) treated with eculizumab or ravulizumab in the intensive or intermediate care unit (ICU/IMC). The primary outcome was the proportion of patients discharged from ICU/IMC within six weeks after C5‐I initiation (German Clinical Trials Registry; DRKS00032104).

**Results:**

Among 17 identified cases, seven had thymoma‐associated MG (TAMG) and 10 had late‐onset MG; median age was 77 years. Five patients required non‐invasive and 12 invasive ventilation; nine underwent tracheotomy. Therapies included IVIg, PLEX, IA, and corticosteroids. Rituximab was added in two cases. Twelve patients received eculizumab and five ravulizumab. Median hospitalization before C5‐I initiation was 32 days (IQR 22–50) and median ICU‐stay was 17 days (IQR 4.5–35) thereafter. Fourteen patients (82%) reached the primary outcome. Two patients died due to bacterial sepsis; no meningococcal infection was observed.

**Conclusion:**

In MC or severe exacerbations insufficiently responsive to standard treatment with IVIg and PLEX/IA, add‐on C5‐I therapy may represent an effective therapeutic approach. Importantly, TAMG, a subtype typically excluded from interventional trials yet often requiring more intensive therapy, also demonstrated clinical improvement. These findings warrant further systematic evaluation of C5‐I as adjunctive therapy for AChR‐ab+ therapy‐refractory MC.

## Graphical abstract

C5‐inhibition may represent an effective adjunctive treatment in therapy‐refractory myasthenic crisis, including thymoma‐associated MG (TAMG).

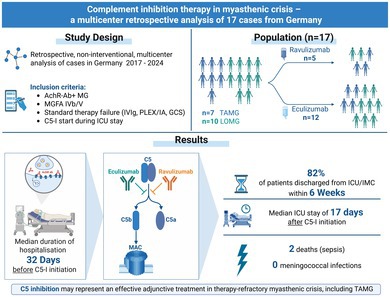

## 1 Introduction

Myasthenia gravis (MG) is a chronic, autoimmune neuromuscular disease characterized by exertion‐dependent and fluctuating muscle weakness. In generalized MG (gMG) any voluntary muscle can be affected, and involvement of the respiratory muscles can lead to life‐threatening myasthenic crisis requiring mechanical ventilation. The Myasthenia Gravis Foundation of America (MGFA) classifies myasthenic crisis as grade V, while a myasthenic exacerbation with impending crisis (MGFA grade IVb) describes a rapid and severe deterioration with pronounced bulbar and/or respiratory weakness, in which progression to crisis within days to weeks is likely but ventilation is not yet required. Although MGFA IVb does not meet the criteria for crisis, it constitutes a severe condition often managed similarly to myasthenic crisis to prevent further deterioration. Although MGFA IVb and V are distinctly defined, the clinical transition between them may occur rapidly, rendering the boundary difficult to delineate in practice. Standard treatment for myasthenic crisis or severe exacerbations includes intravenous immunoglobulins (IVIg), plasmapheresis (PLEX) or immunoadsorption (IA) usually combined with high‐dose corticosteroids [1, 2, 3]. Despite these interventions, in up to 20% of cases, prolonged ventilation may become necessary [4]. In selected refractory cases, escalating immunomodulating therapies such as rituximab and eculizumab have been reported [5, 6, 7, 8]. More recently, also ravulizumab [8, 9, 10], zilucoplan [11, 12] and the FcRn‐inhibitor efgartigimod [13, 14, 15, 16] have been considered as add‐on treatment during therapy‐refractory myasthenic crisis. While rituximab requires at least three months to achieve therapeutic efficacy, complement inhibitors (C5‐I) and FcRn‐inhibitors induced clinically meaningful improvement within weeks of treatment initiation in phase 3 clinical trials [17, 18, 19, 20]. In recent years, the spectrum of available and approved add‐on therapies for patients with gMG and a high disease activity has broadened to two FcRn‐inhibitors and three C5‐I [17].

The first C5‐I with approval for therapy of patients with acetylcholine‐receptor‐antibody (AChR‐ab) positive (treatment‐refractory) gMG was eculizumab. The approval was based on the REGAIN trial results [21]. Ravulizumab, an engineered derivative of eculizumab with prolonged half‐life, was approved next based on the results of the CHAMPION MG trial [22]. Zilucoplan, a macrocyclic peptide with daily subcutaneous application, was approved most recently based on the results of the RAISE trial [23]. Pivotal trials of all three agents systematically excluded patients with myasthenic crisis or severe unstable disease (MGFA class IVb–V), substantially limiting evidence in this high‐risk population. To date, 23 cases of C5‐I use in cases of myasthenic crisis or impending myasthenic crisis have been reported: 17 cases with use of eculizumab [5, 6, 7, 8, 24, 25, 26, 27, 28], three cases with use of ravulizumab [8, 9, 10] and three cases with use of zilucoplan [11, 12].

The aim of this study was to determine the potential beneficial effects of C5‐I as add‐on treatment for patients with gMG refractory to the standard treatment of myasthenic crisis (termed therapy‐refractory myasthenic crisis) in a systematically collected retrospective series of cases from Germany.

## 2 Methods

### 2.1 Ethics Approval and Study Registration

The study was conducted according to the Declaration of Helsinki and approved by the Ethics Committee of the Charité—Universitätsmedizin Berlin (EA1/109/23). Due to the retrospective nature of the study, written informed consent was not necessary based on local regulation (“Berliner Landeskrankenhausgesetz”). This article follows the Strengthening the Reporting of Observational Studies in Epidemiology (STROBE) reporting guidelines. The study was registered in the German Clinical Trials Registry (DRKS00032104).

### 2.2 Study Design and Definitions

This was a retrospective, non‐interventional, multicentre analysis of cases in Germany between 2017 and 2024. Inclusion criteria were a myasthenic crisis or an impending myasthenic crisis involving bulbar symptoms (MGFA IVb/V) in patients with AChR‐ab positive gMG who started eculizumab or ravulizumab in the intensive care unit (ICU) or intermediate care unit (IMC). The primary outcome was the proportion of patients discharged from ICU/IMC within six weeks after initiation of C5‐I therapy. The timeframe of six weeks was predefined after expert consensus as a realistic aim for clinical stabilization after new treatment initiation.

Myasthenic crisis was defined as a life‐threatening exacerbation of myasthenic symptoms (with bulbar or general weakness) requiring intubation and invasive ventilation, classified as MGFA V. Severe exacerbation with impending myasthenic crisis was defined as the rapid clinical worsening of MG involving bulbar and/or respiratory symptoms potentially leading to non‐invasive ventilatory support, classified as MGFA IVb. “Crisis‐like course” before admission to ICU was defined as a disease course with more than one admission to hospital due to exacerbation of myasthenic symptoms with necessity of therapeutic intervention (e.g., IVIg), with or without the need for ventilatory support, within the three months preceding the index crisis with use of C5‐I.

### 2.3 Case Identification and Data Collection

Cases were identified from 18 tertiary MG centres of excellence (German: integrierte Myastheniezentren, abbr.: iMZs) in Germany, which are certified by the German myasthenia gravis association. Cases fulfilling inclusion criteria were then analysed retrospectively including data before the myasthenic crisis with the use of C5‐I, during the myasthenic crisis and a follow‐up of up to 6 months after the myasthenic crisis.

MGFA classification [29] used as current status score, Myasthenia Gravis Activities of Daily Living (MG‐ADL, 0–24 point score) [30], Quantitative Myasthenia Gravis score (QMG, 0–39 point score) [31], Besinger Score (0–3 point score) [32], and vital capacity measurements were collected retrospectively if they had been performed and documented.

### 2.4 Statistical Analysis

The statistical analyses were performed using R software (version 4.2.2) and R Studio software (version 2023.03.1 build 446). Descriptive statistics (mean, standard deviation, median, interquartile range, absolute and relative frequencies) were applied to summarize patient characteristics and therapies where applicable.

## 3 Results

We identified 19 cases from six participating centres in Germany between 2017 and 2023, in which C5‐I therapy was initiated during a myasthenic crisis or impending myasthenic crisis that required IMC/ICU treatment. Two cases were excluded for not meeting all inclusion criteria (Supplemental Figure 1). Of the remaining 17 patients, 12 received eculizumab and five ravulizumab. One of the eculizumab‐treated patients had previously been published as a single case report [7].

### 3.1 Characteristics of MG before Index Crisis

Of the 17 cases included, 7 (41%) were female and the median age was 77 years (IQR 67–83). Ten patients (59%) had late‐onset MG (LOMG) and 7 (41%) thymoma‐associated MG (TAMG). Two TAMG patients were younger than 50 years. All 17 patients were AChR‐ab‐positive and 11 (65%) were positive for titin‐antibodies. Median time from diagnosis to index crisis was 0.6 years (IQR 0.0–6.3) with 10 (59%) experiencing the index crisis within the first year after diagnosis and 3 (18%) diagnosed during the index crisis. Seven patients (41%) had undergone thymectomy, of which six had a thymoma. One additional thymoma was diagnosed by biopsy only, bringing the total number of TAMG in the cohort to seven. Patients had a median number of five comorbidities and six non‐MG‐comedications (Table 1). Patients in the ravulizumab subgroup were slightly younger.

**TABLE 1:** Demographics, characteristics of myasthenia gravis, MG Medication before index crisis, previous myasthenic crisis, previous rescue therapies.

	Overall *N* = 17	Eculizumab *N* = 12	Ravulizumab *N* = 5
Female sex, *n* (%)	7 (41%)	4 (33%)	3 (60%)
Age at diagnosis (yrs), median (iqr)	72.0 (59.5; 80.3)	71.0 (61.8; 80.9)	72.0 (37.8; 73.0)
Age at myasthenic crisis (yrs), median (iqr)	77.0 (67.0; 83.0)	81.0 (69.5; 84.5)	73.0 (46.0; 77.0)
EOMG, *n* (%)	0 (0%)	0 (0%)	0 (0%)
LOMG, *n* (%)	10 (59%)	8 (67%)	2 (40%)
TAMG, *n* (%)	7 (41%)	4 (33%)	3 (60%)
BMI in kg/m^2^, median (IQR)	29.0 (27.5;33.7)	27.6 (26.4;29.5)	31.5 (29.1;33.8)
AChR‐ab positive, *n* (%)	17 (100%)	12 (100%)	5 (100%)
Titin‐ab positive, *n* (%)	11 (65%)	8 (67%)	3 (60%)
Pathologic decrement in RNS, *n* (%)	10 (59%)	6 (50%)	4 (80%)
Pathologic single‐fibre EMG, *n* (%)	4 (24%)	2 (17%)	2 (40%)
Duration first symptoms to diagnosis (days), median (IQR)	31.0 (0.0; 118.0)	31.0 (0.0; 96.5)	30.0 (0.0; 304.0)
Duration from diagnosis to index crisis (years), median (IQR)	0.6 (0.0; 6.3)	0.3 (0.0; 4.6)	0.7 (0.0; 6.6)
Diagnosis during index crisis, *n* (%)	3 (18%)	2 (17%)	1 (20%)
Index crisis within first year after diagnosis, *n* (%)	10 (59%)	7 (58%)	3 (60%)
Symptoms at disease onset, *n* (%) (multiple choices possible)			
Ocular	8 (47%)	5 (42%)	3 (60%)
Generalized	8 (47%)	6 (50%)	2 (40%)
Faciopharyngeal symptoms	11 (65%)	8 (67%)	3 (60%)
Myasthenic crisis as first symptom	2 (12%)	1 (8%)	1 (20%)
Thymectomy, *n* (%)	7 (41%)	4 (33%)	3 (60%)
Thymectomy within 2 years. after diagnosis, *n* (%)	6 (86%)	3 (75%)	3 (100%)
Histopathology of thymectomy, *n* (%)			
Normal	1 (14%)	1 (25%)	0 (0%)
Thymoma	6 (86%)	3 (75%)	3 (100%)
Thymic biopsy, *n* (%)	1 (6%)	1 (8%)	0 (0%)
Histopathology of thymic biopsy, *n* (%)			
Thymoma	1 (100%)	1 (100%)	0 (0%)
Total number of thymomas^b^, *n* (%)	7 (41%)	4 (33%)	3 (60%)
Comorbidities (number of 13 categories^a^), median (IQR)	5.0 (3.0;6.0)	4.0 (2.5;5.5)	5.0 (5.0;6.0)
Comorbidities (≥ 3 of 13 categories)	14 (82%)	9 (75%)	5 (100%)
Number of non‐MG comedication, median (IQR)	6.0 (4.0, 9.0)	6.0 (4.5, 8.5)	4.0 (2.0, 9.0)
MG medication before index crisis			
Pyridostigmine, *n* (%)	16 (94%)	11 (92%)	5 (100%)
Pyridostigmine dosage in mg, median (iqr)	240 (165;435)	285 (90;473)	200 (180;300)
Prednisolone, *n* (%)	15 (88%)	10 (83%)	5 (100%)
Prednisolone dosage in mg, median (iqr)	22.5 (16.3, 40.0)	20.0 (15.0, 40.0)	25.0 (20.0, 60.0)
Historical prednisolone therapy, *n* (%)	1 (6%)	1 (8%)	0 (0%)
Azathioprine, *n* (%)	6 (35%)	4 (33%)	2 (40%)
Azathioprine dosage in mg, median (iqr)	100 (62.5, 138)	100 (81.3, 113)	100 (75.0, 125)
Historical azathioprine therapy, *n* (%)	3 (18%)	2 (17%)	1 (20%)
MMF, *n* (%)	3 (18%)	2 (17%)	1 (20%)
MMF dosage in mg, median (iqr)	1500 (1500, 2000)	2000 (1750, 2250)	1500 (1500, 1500)
Historical MMF therapy, *n* (%)	0 (0%)	0 (0%)	0 (0%)
Methotrexate (MTX), *n* (%)	0 (0%)	0 (0%)	0 (0%)
Historical MTX therapy, *n* (%)	1 (6%)	1 (8%)	0 (0%)
Cyclosporine A (CSA), *n* (%)	0 (0%)	0 (0%)	0 (0%)
Historical CSA therapy, *n* (%)	0 (0%)	0 (0%)	0 (0%)
Number of previous myasthenic crises, *n* (%)			
0	6 (35%)	4 (33%)	2 (40%)
1	5 (29%)	3 (25%)	2 (40%)
2	4 (24%)	3 (25%)	1 (20%)
Missing information	2 (12%)	2 (17%)	0 (0%)
IVIg within 12 months before index crisis, *n* (%)			
Yes, as rescue therapy	8 (47%)	5 (42%)	3 (60%)
Yes, regularly	2 (12%)	1 (8%)	1 (20%)
No	6 (35%)	5 (42%)	1 (20%)
Missing information	1 (6%)	1 (8%)	0 (0%)
Plasmapheresis/Immunoadsorption within 12 months before index crisis, *n* (%)	4 (24%)	3 (25%)	1 (20%)

Abbreviations: ab: antibody; AChR: acetylcholine‐receptor; BMI: body‐mass index; CSA: cyclosporine A; EMG: electromyography; EOMG: early‐onset myasthenia gravis; IVIg: intravenous immunoglobulins; IQR: inter‐quartile range; LOMG: late‐onset myasthenia gravis; mg: milligram; MMF: mycophenolate‐mofetil; MTX: methotrexate RNS: repetitive nerve stimulation; TAMG: thymoma‐associated myasthenia gravis; yrs.: years.

^a^ The 13 categories of comorbidities were: cardiovascular diseases, pulmonary diseases, renal diseases, gastrointestinal diseases, metabolic diseases, autoimmune diseases, oncological diseases, neurological diseases, psychiatric diseases, addiction‐related health problems, dermatological diseases, ophthalmological diseases, and musculoskeletal diseases.

^b^ The total number of thymomas consists of 6 thymomas that were thymectomized and 1 thymoma that was diagnosed by biopsy only. This patient died during the crisis and therefore no thymectomy was performed.

At hospitalization for the index crisis, 16 patients (94%) received pyridostigmine and 15 (88%) prednisolone. Long‐term immunosuppressants were used in nine patients (53%): six patients (35%) received azathioprine and three (18%) mycophenolate‐mofetil (MMF). Nine patients (53%) had experienced at least one previous myasthenic crisis, and four (27%) had had two previous crises. Ten (59%) had received IVIg and four (24%) PLEX or IA within 12 months before the index crisis, indicating a high disease activity in the cohort (Table 1).

### 3.2 Characteristics of Myasthenic Crisis with Use of C5‐I

Infections were the most frequent trigger of the index crisis including pneumonia, Clostridium‐associated enteritis, sepsis, COVID‐19, Influenza, and varicella zoster virus (VZV) encephalitis. The most common symptoms during myasthenic crisis were masticatory and/or pharyngeal weakness (94%), dyspnoea (82%), and generalized limb weakness (82%) (Table 2).

**TABLE 2:** Characteristics of myasthenic crisis and outcomes.

	Overall *N* = 17	Eculizumab *N* = 12	Ravulizumab *N* = 5
Triggers of myasthenic crisis (multiple choices possible), *n* (%)			
Infection	10 (59%)	7 (58%)	3 (60%)
Medication^a^	1 (6%)	1 (8%)	0 (0%)
Surgery/anesthesia	1 (6%)	1 (8%)	0 (0%)
Other triggers^b^	4 (24%)	3 (25%)	1 (20%)
Unknown	3 (18%)	2 (17%)	1 (20%)
Myasthenic symptoms during crisis (multiple choices possible), *n* (%)			
Generalized limb weakness	14 (82%)	10 (83%)	4 (80%)
Weakness in neck and/or axial muscles	7 (41%)	4 (33%)	3 (60%)
Weakness in masticatory and/or pharyngeal muscles	16 (94%)	12 (100%)	4 (80%)
Slurred speech	9 (53%)	6 (50%)	3 (60%)
Dyspnoea	14 (82%)	10 (83%)	4 (80%)
Ptosis	5 (29%)	4 (33%)	1 (20%)
Double vision	7 (41%)	5 (42%)	2 (40%)
Duration of “crisis‐like course”^c^ before admission to ICU (days), median (IQR)	11.0 (0.0;70.0)	42.0 (1.5;85.0)	0.0 (0.0;8.0)
Min, Max	0.0, 147.0	0.0, 147.0	0.0, 137.0
Duration of “crisis‐like course”^c^ before admission to ICU > 1 week, *n* (%)	10 (59%)	8 (67%)	2 (40%)
Duration of hospitalization (days), median (IQR)	51.0 (44.0;97.0)	84.5 (48.5;116.5)	44.0 (35.0;50.0)
Min, Max	18.0, 327.0	30.0, 327.0	18.0, 65.0
Duration ICU stay (days), median (IQR)	48.5 (34.5;83.5)	76.0 (34.0;107.0)	44.0 (35.0;48.0)
Min, Max	13.0, 327.0	17.0, 327.0	13.0, 50.0
Duration of hospitalization (days) before start of ECU/RAVU, median (IQR)	32.0 (22.0;50.0)	32.5 (26.0;51.0)	22.0 (14.0;34.0)
Duration ICU stay (days) since start of ECU/RAVU, median (IQR)	17.0 (4.5;35.0)	18.0 (8.0;44.0)	3.0 (1.0;22.0)
Ventilation support and PEG			
Only non‐invasive ventilation support	5 (29%)	2 (17%)	3 (60%)
Invasive ventilation	12 (71%)	10 (83%)	2 (40%)
Duration of invasive ventilation (days); Median (IQR)	41.0 (13.0;101.0)	62.0 (17.0;136.0)	3.0 (3.0;3.0)
Invasive ventilation > 15 days, *n* (%)	6 (67%)	6 (75%)	0 (0%)
Tracheotomy	9 (75%)	8 (80%)	1 (50%)
PEG	6 (35%)	4 (33%)	2 (40%)
Transferral from ICU to			
Normal ward	8 (47%)	6 (50%)	2 (40%)
IMC	1 (6%)	1 (8%)	—
Directly to rehabilitation/weaning unit	6 (35%)	3 (25%)	3 (60%)
Death during ICU stay	2 (12%)	2 (17%)	—
Primary outcome			
Proportion of patients leaving ICU/IMC within 42 days = 6 weeks after initiation of C5‐I, *n* (%)	14 (82%)	9 (75%)	5 (100%)
Proportion of patients leaving ICU/IMC within 70 days = 10 weeks after initiation of C5‐I, *n* (%)	15 (88%)	10 (83%)	5 (100%)

Abbreviations: Ecu: eculizumab; ICU: intensive care unit; IMC: intermediate care unit; IQR: inter‐quartile range; PEG: percutaneous; Ravu: ravulizumab.

^a^ Use of fentanyl‐patch.

^b^ Other triggers for crisis named were thymoma, recurrent thymoma, temporal correlation with COVID‐19‐vaccination.

^c^ “Crisis‐like course of disease” was defined as disease course with more than one admission to hospital due to exacerbation of myasthenic symptoms with necessity of therapeutic intervention (e.g., IVIg) in the three months leading up to the index crisis with use of C5‐I.

**FIGURE 1:**
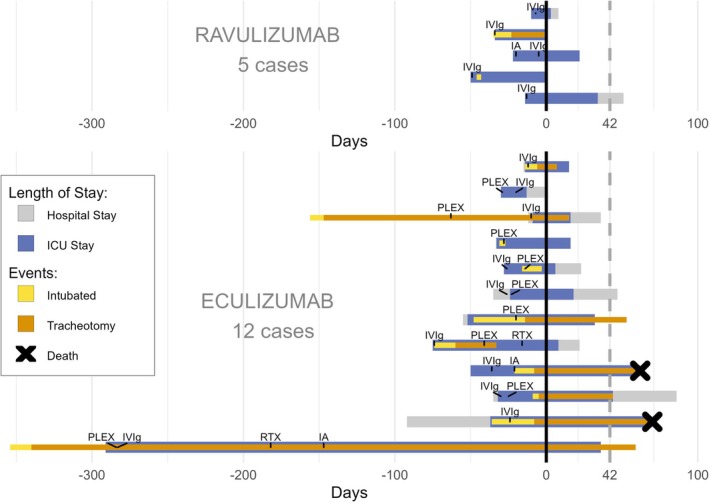
Duration of hospitalization, ICU‐stay and invasive ventilation as well as therapies administered relative to start of C5‐inhibitor for all 17 cases. Primary outcome marked with dashed grey line at day 42. Day Zero is the day of C5‐I initiation. In those patients where the duration of invasive ventilation (intubated as yellow bar and tracheotomized as orange bar) exceeds the hospitalization, the patients were intubated/tracheotomized at an external hospital and then transferred to the respective participating centre in the study. IA: Immunoadsorption; ICU: Intensive care unit; IVIg: Intravenous immunoglobulins; PLEX: Plasmapheresis; RTX: Rituximab.

**FIGURE 2:**
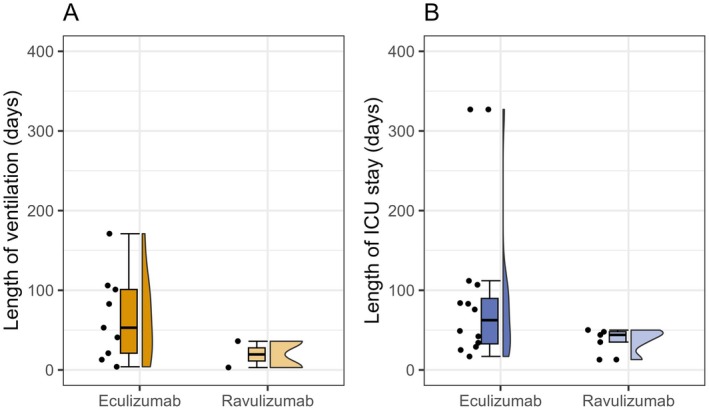
Comparison of length of ventilation (A) and length of ICU stay (B) between eculizumab and ravulizumab subgroup. ICU: Intensive care unit.

During the index crisis, patients spent a median of 48.5 days (IQR 34.5–83.5) in ICU/IMC with marked heterogeneity across the cohort: ICU/IMC stay was notably longer in the eculizumab subgroup than in the ravulizumab subgroup (76 days (IQR 34–107) versus 44 days (IQR 35–48)) with a maximum of 327 days (Table 2 and Figure 1). More than half of the patients (59%) had a “crisis‐like course” for over a week before ICU admission. Median total hospitalization was 51 days, again longer in the eculizumab than in the ravulizumab subgroup (84.5 vs. 44 days). Median hospitalization before C5‐I initiation was 32 days (IQR 22–50), and median length of ICU/IMC stay was 17 days (IQR 4.5–35) after C5‐I initiation. Again, duration was notably longer in the eculizumab subgroup, consistent with their overall longer ICU stay. Non‐invasive ventilation was sufficient in 5 (29%) cases. 12 (71%) required invasive ventilation, including nine tracheotomies. Six patients required invasive ventilation for more than 15 days; all in the eculizumab subgroup. A percutaneous endoscopic gastrostomy (PEG) was needed in six cases due to severe, prolonged dysphagia (Table 2, Figures 1 and 2).

### 3.3 Transferral from ICU, Primary Outcome and Survival

Within the cohort, eight patients were transferred to a normal ward, one to an IMC and six directly to a rehabilitation or weaning facility. Two patients died during the ICU‐stay. These two patients are described in more detail below. Of the 15 survivors, 14 (82%) achieved the primary outcome—discharge from ICU/IMC within six weeks after C5‐I initiation (Table 2 and Figure 1).

Both patients, who died, were male, of similar age close to the cohort median of 77 years and had three to four comorbidities. One patient was diagnosed with TAMG during the index crisis; thymoma was confirmed by biopsy after chest CT but no thymectomy was performed. The other patient had a long‐standing MG diagnosis (> 10 years) and had not been thymectomized due to LOMG and no presence of a thymoma in chest CT. The index crisis was triggered by surgery and an infection. Both received prednisolone and IVIg, one additionally immunoadsorption, and both eculizumab. Deaths occurred 9 and 10 weeks after initiation of eculizumab due to complications: One patient developed a myocardial infarction with AV‐block requiring temporal pacing, followed by a sepsis and death after a palliative care decision. The other patient developed a septic shock due to vancomycin‐resistant enterococcus, resulting in death.

### 3.4 Therapies and Complications during Myasthenic Crisis

During the myasthenic crisis, 12 patients (71%) received intravenous pyridostigmine or neostigmine. All 17 patients were treated with prednisolone (range 15–1000 mg/day; median dose 30 mg). Only IVIg were administered in six patients; only PLEX in two; and both IVIg and PLEX and/or IA in nine patients. Rituximab (RTX) was initiated in two patients before C5‐I initiation (16 and 182 days before C5‐I; Figure 1). Further escalation therapies included high dose corticosteroids (500 and 1000 mg/day), and one patient received five cycles of bortezomib before eculizumab (Table 3).

**TABLE 3:** Therapies and complications during myasthenic crisis.

	Overall *N* = 17	Eculizumab *N* = 12	Ravulizumab *N* = 5
MG Medication during crisis			
Pyridostigmine orally dosage in mg, median [IQR]	420 (270;510)	420 (315;510)	300 (240;420)
Pyridostigmine or neostigmine intravenously, *n* (%)	12 (71%)	10 (83%)	2 (40%)
Prednisolone highest dose in mg during crisis, median [IQR]	30.0 (20.0;60.0)	25.0 (20.0;52.5)	60.0 (30.0;60.0)
Min, Max	15.0, 1000	15.0, 1000	20.0, 60.0
IVIg	15 (88%)	10 (83%)	5 (100%)
IVIg cumulative dose in g, median [iqr]	180 (150;200)	170 (150;184)	200 (150;240)
IVIg days, median [IQR]	5.00 [5.00, 6.00]	5.00 [5.00, 5.75]	5.00 [4.00, 6.00]
Plasmapheresis (PLEX)	9 (53%)	9 (75%)	0 (0%)
Number of cycles, median [IQR]	7.50 [5.75;11.0]	7.50 [5.75;11.0]	—
Number of cycles, Min, Max	5.00, 51.0	5.00, 51.0	—
Immunoadsorption (IA)	3 (18%)	2 (17%)	1 (20%)
Number of cycles, median [IQR]	5.00 [4.00;7.00]	4.50 [4.25;4.74]	7.00 [7.00;7.00]
Number of cycles, Min, Max	4.00;7.00	4.00;5.00	7.00;7.00
Only IVIg	6 (35%)	2 (17%)	4 (80%)
IVIG and PLEX	6 (35%)	6 (50%)	0 (0%)
Only PLEX	2 (12%)	2 (17%)	0 (0%)
IVIg and IA	2 (11.8%)	1 (8%)	1 (20%)
IVIg and PLEX and IA	1 (6%)	1 (8%)	0 (0%)
Rituximab started during crisis^a^	2 (12%)	2 (17%)	0 (0%)
Other escalation therapies^b^	3 (18%)	3 (25%)	0 (0%)
Complications			
Cases with (severe) complications, *n* (%)	12 (71%)	8 (67%)	4 (80%)
Number of (severe) complications per patient; median [IQR]	2 [1;3]	3 [1;3.5]	1 [1;1]
Min, Max	0; 6	0; 6	0; 2
Which complications (multiple choices possible), *n* (%)			
any infection	10 (59%)	7 (58%)	3 (60%)
Pneumonia	7 (41%)	5 (42%)	2 (40%)
Urinary tract infection	5 (29%)	4 (33%)	1 (20%)
Sepsis	4 (24%)	4 (33%)	0 (0%)
Upper respiratory tract infection	2 (12%)	2 (17%)	0 (0%)
Fungal disseminated infection	1 (6%)	1 (8%)	0 (0%)
Localized Herpes Virus infection	1 (6%)	1 (8%)	0 (0%)
any cardiac complication	4 (24%)	4 (33%)	0 (0%)
Asystole/severe cardiac arrhythmias	3 (18%)	3 (25%)	0 (0%)
Cardiac infarction	2 (12%)	2 (17%)	0 (0%)
Resuscitation	1 (6%)	1 (8%)	0 (0%)
Hypertensive crisis	3 (18%)	2 (17%)	1 (20%)
Pleural effusion	2 (12%)	1 (8%)	1 (20%)
Pneumothorax	1 (6%)	1 (8%)	0 (0%)
GI‐bleeding	1 (6%)	1 (8%)	0 (0%)

Abbreviations: Ecu: eculizumab; GI: gastro‐intestinal; IA: immunoadsorption; IMC: intermediate care unit; IVIg: intravenous immunoglobulins; IQR: inter‐quartile range; Max: maximum; Min: minimum; PEG: percutaneous; PLEX: plasmapheresis; Ravu: ravulizumab.

^a^ Both patients who received rituximab received it before start of eculizumab.

^b^ Other escalation therapies were high‐dose methylprednisolone (2 cases) and Bortezomib (5 cycles; given before eculizumab).

Twelve patients (71%) developed at least one severe complication during their ICU/IMC stay and before C5‐I initiation with a median of two (IQR 1–3) per patient. The most frequent were infections (59%), primarily pneumonia (41%), urinary tract infections (29%), and sepsis (24%). Cardiac events occurred in 4 patients (24%). No case of meningococcal infection was reported (Table 3).

### 3.5 Vaccination Against Meningococcus and Available Scores

Sixteen patients had been vaccinated against meningococcus ACWY and B before starting C5‐I therapy. Vaccination status was unknown in one individual. However, this patient received an antibiotic prophylaxis and survived. Four patients were fully vaccinated, having received at least one ACWY and at least two B doses≥two weeks before C5‐I initiation. In 12 patients, vaccination was completed less than two weeks before C5‐I initiation, and of these, 11 patients received an antibiotic prophylaxis. Prophylaxis given until two weeks after vaccination included ceftriaxone, azithromycin, rifampicin, ampicillin, amoxicillin or penicillin G (Supplementary Table 1).

**FIGURE 3:**
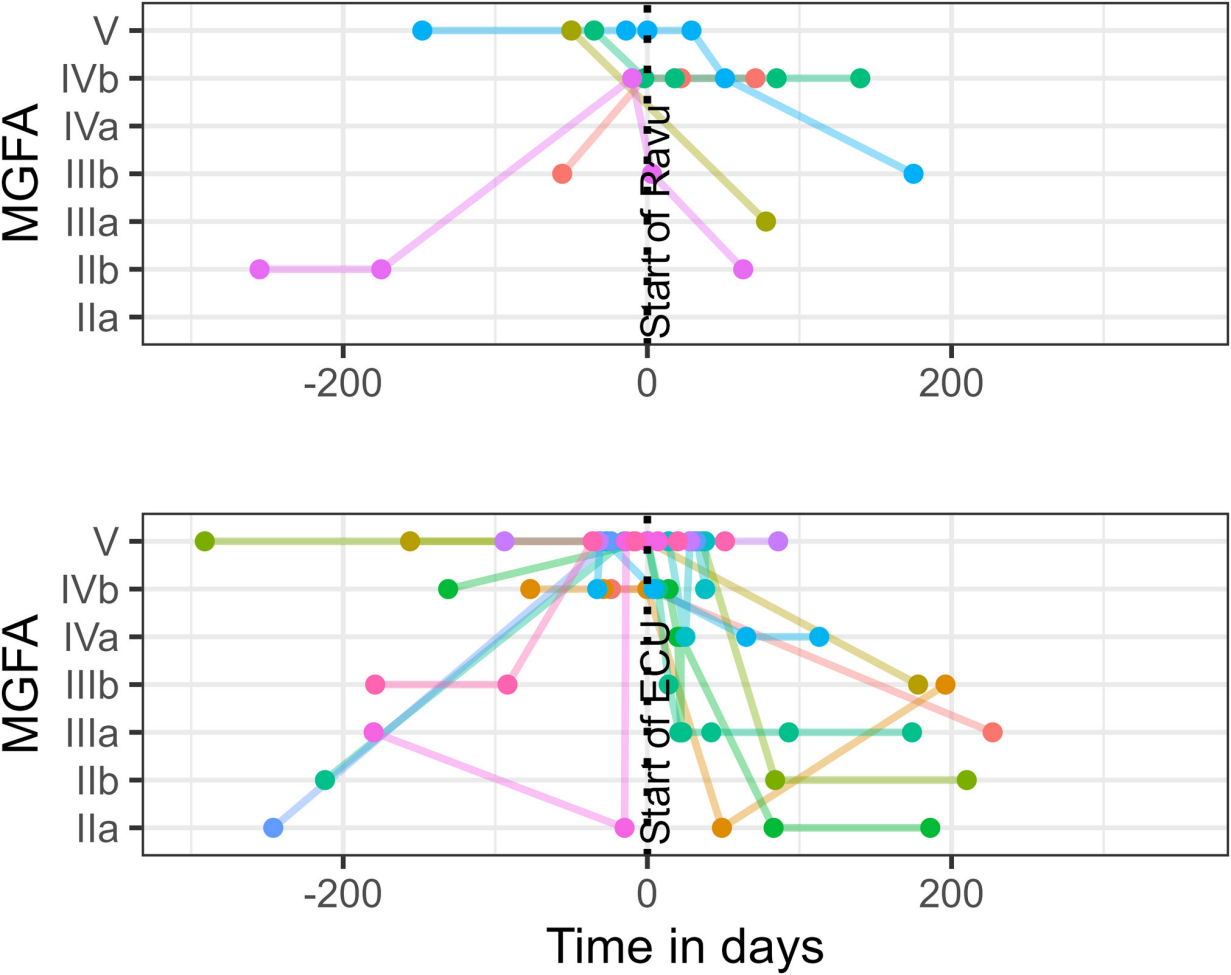
MGFA classification used as current status score over time for ravulizumab and eculizumab. Ecu: Eculizumab; Ravu: Ravulizumab.

Availability of scores varied considerably across patients and centres due to the retrospective study design. All 17 patients had at least three MGFA assessments documented as current status (Figure 3). Other scores (MG‐ADL, QMG, Besinger and vital capacity) were available less frequently, in some cases only once (Supplementary Table 2). No quality‐of‐life scores (e.g., MG‐QoL15 or MG‐QoL15r) were available.

### 3.6 Long‐Term Follow‐Up and Corticosteroid Dosage After the Index Crisis

**FIGURE 4:**
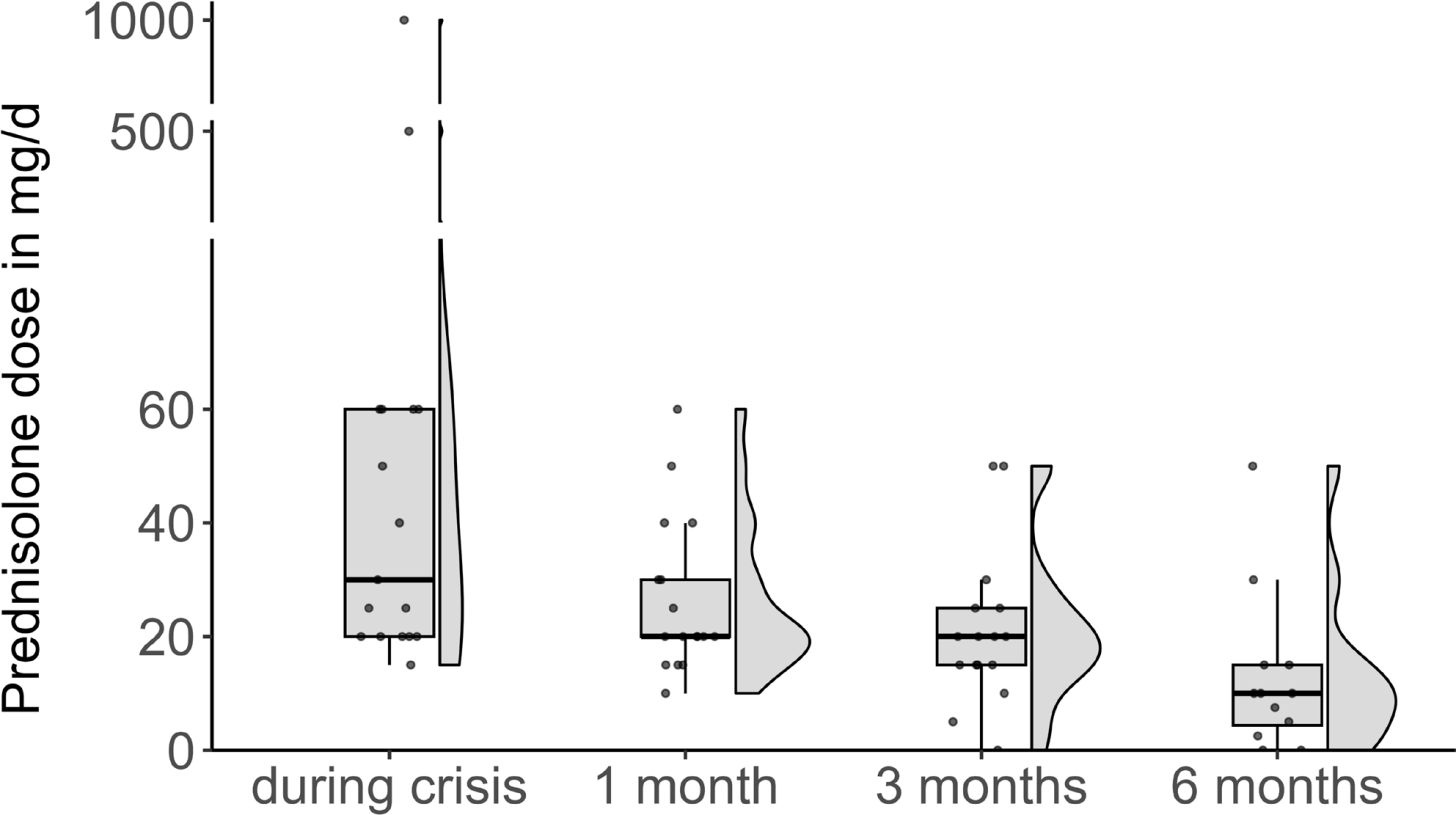
Raincloud plots of prednisolone dose during six months of follow‐up after start of C5‐Inhibitor.

Patients were followed for up to six months after the index crisis. During follow‐up, three patients required additional IVIg treatments: two within one month after starting eculizumab and one within three months after starting ravulizumab. The prednisolone dose was successfully tapered in most patients, from a median daily dose of 30 mg [IQR 20–60] during crisis to 20 mg [IQR 20–30], 20 mg [IQR 15–22.5] and 10 mg [IQR 3.1–13.8] at one, three, and six months respectively (Figure 4).

Of 17 patients, 11 (65%) were still under care at the participating centre. Two (12%) had died during hospitalization as reported above. Follow‐up data was available for three months in two (12%) cases and for six months in another two cases (12%). Among the 11 patients with available long‐term follow‐up, seven belonged to the eculizumab subgroup and four to the ravulizumab subgroup. In the eculizumab subgroup, one had remained on eculizumab, five had switched to ravulizumab for convenience, and one to the FcRn‐inhibitor efgartigimod. All four ravulizumab patients with long‐term follow‐up continued ravulizumab therapy at the time of data collection.

## 4 Discussion

In this retrospective cohort of patients with therapy‐refractory myasthenic crisis or severe exacerbation with impending crisis, in whom standard therapy with IVIg and/or PLEX failed to achieve sufficient clinical stabilization, treating physicians initiated add‐on complement C5‐I to facilitate ventilatory weaning and enable recovery. A total of 82% of patients achieved the predefined primary outcome of ICU discharge within six weeks after initiation of C5‐I, with a substantially shorter median time to discharge of 17 days. These findings provide additional real‐world evidence that add‐on C5‐I therapy may offer an effective and reasonably safe escalation option at least when conventional crisis management proves insufficient.

Both eculizumab and ravulizumab have previously been shown to be effective in achieving relevant improvements in gMG [21, 22, 33]. However, their therapeutic value during myasthenic crisis remains uncertain and is largely informed by real‐world data, given the exclusion of crisis cases from pivotal trials. Even in large registry cohorts—such as the Myasthenia Gravis SPOTLIGHT Registry [34]—myasthenic crisis representation remains sparse, with only one out of 189 patients reported as MGFA class V before eculizumab initiation.

Myasthenic crisis is a rare but potentially life‐threatening complication of gMG and may lead to lengthy ICU stays with prolonged ventilation. In a large retrospective cohort from the United States, the mortality for patients with a myasthenic crisis was 4.5% [35] and in a prospective study from China in‐hospital mortality from myasthenic crisis was 2.6% [36]. However, in a more recent and prospective series of 250 cases with myasthenic crisis, the mortality rate was 12% and was associated with the number of comorbidities and the number of complications [4]. The number of complications usually correlates with the length of the ICU stay and with the number of ventilation days and all three influence the probability of survival [36, 37, 38]. In our cohort, 71% of patients experienced at least one complication with infections being the most frequent (59%). The rate of infections in our cohort was therefore higher than in the phase‐3 trials and open‐label extension phases for eculizumab and ravulizumab, where the total rate of infections was between 15% and 49% [19, 21, 22, 39]. This is well explained by the special setting of our cohort in the ICU. Mortality in our cohort was 12% and therefore not higher than in the larger cohort of 250 cases of myasthenic crisis without C5‐I therapy [4]. The mortality in other published cases with use of C5‐I during an (impending) myasthenic crisis was lower, with only one death [26] among 22 reported cases [5, 6, 8, 9, 10, 11, 12, 24, 25, 26, 27, 28], which was reported as part of a small case series (Table 4). Likely, a reporting bias exists with lower probability of reporting cases with negative outcome. This emphasizes the value of a systematically collected larger cohort like ours, which facilitates reporting of cases with an unfavourable outcome.

**TABLE 4:** Comparison of our 17 cases with 22 cases in the literature.

	Literature Review *N* = 22	Our cohort *N* = 17^a^
Age (yrs); median [IQR]	66 [47; 73]	77.0 (67;83)
Female sex, *n* (%)	12 (57%)	7 (41%)
MG subtype		
AChR‐ab+, *n* (%)	21 (95%)	17 (100%)
EOMG, *n* (%)	3 (14%)	0 (0%)
LOMG, *n* (%)	13 (59%)	10 (59%)
TAMG, *n* (%)	6 (27%)	7 (41%)
Cause of (impending) crisis		
infection, *n* (%)	4 (18%)	10 (59%)
surgery, *n* (%)	3 (14%)	1 (6%)
other, *n* (%)	1 (5%)	5 (29%)
unknown, *n* (%)	14 (64%)	3 (18%)
MC with invasive ventilation, *n* (%)	18 (82%)	12 (71%)
tracheotomy, *n* (%)	11 (50%)	9 (53%)
Impending MC, *n* (%)	4 (18%)	5 (29%)
Rescue therapies, *n* (%)		
steroids	19 (86%)	17 (100%)
IVIg	20 (91%)	15 (88%)
PLEX/IA	15 (68%)	11 (65%)
Other escalation therapies		
Rituximab	4 (18%)	2 (12%)
Efgartigimod	1 (5%)	—
C5‐inhibitor administered		
Eculizumab	16 (73%)	12 (71%)
Ravulizumab	3 (13%)	5 (29%)
Zilucoplan	3 (13%)	—
Complications, *n* (%)		
infections	9 (41%)	10 (59%)
cardiac complication	3 (14%)	4 (24%)
other^b^	4 (18%)	7 (41%)
Outcome		
Follow‐up period	2 months to 1 year	3 to 6 months
Positive outcome^c^	19 (86%)	15 (88%)
Deaths	1 (5%)	2 (12%)

Abbreviations: AChR‐ab+: acetylcholine‐receptor‐antibody positive; EOMG: early‐onset myasthenia gravis; IVIg: intravenous immunoglobulins; IQR: inter‐quartile range; LOMG: late‐onset myasthenia gravis; MC: myasthenic crisis; MG: myasthenia gravis; TAMG: thymoma‐associated myasthenia gravis; yrs.: years.

^a^ One of the 17 cases in our series has been published previously as a single case report; this case is reported within our retrospective case series in this table [7].

^b^ Other complications were: Allergic reaction to IA, unresectable thymoma treated with radiotherapy, intestinal perforation, adrenal insufficiency.

^c^ Positive outcome was defined as successful weaning from ventilation support and an improvement in MG symptoms.

Furthermore, within the follow‐up period of six months after the index crisis and under continued add‐on therapy with the C5‐inhibitor, the corticosteroid dose could be successfully reduced in most cases of our cohort. This is an important observation, given the long‐term deleterious side effects of continued corticosteroid therapy with a daily dose of more than 5 mg. Similar data on the steroid‐sparing effect of eculizumab and ravulizumab come from several real‐world data cohorts [40, 41, 42, 43, 44].

Importantly, seven patients with TAMG also showed a positive response. TAMG represents a subtype of gMG that often not only requires more intensive treatment but also is often excluded from interventional studies. Patients with TAMG tend to have more severe myasthenic symptoms at a younger age, a worse prognosis and have a higher risk of suffering myasthenic crisis [38, 45]. The positive response of this subgroup in our cohort is in line with a small observational study on the use of eculizumab in 22 patients with TAMG from China [46], where the majority achieved a reduction of disease burden and of the corticosteroid dose. Moreover, in two case reports, C5‐I therapy was used as a successful bridging to further thymoma therapy in patients with TAMG [24, 26]. However, two of the three deaths that occurred over all 39 cases of C5‐I therapy during (impending) myasthenic crisis concerned patients with TAMG. This highlights the potential severity of TAMG as a paraneoplastic condition whose outcome heavily depends on the successful removal of the thymoma [36, 45].

## 5 Limitations

The main limitations of this study are the retrospective design, the limited number of cases, the heterogeneity of available data and the lack of a control group. Since all patients received standard crisis therapies before C5‐I initiation, often sequentially, the effects of IVIg and/or PLEX cannot be clearly disentangled from those attributable to C5‐I treatment. In this cohort, complement inhibition was used exclusively as escalation therapy in patients who continued to require intensive care after an insufficient response to standard treatments, introducing a strong selection bias (confounding by indication). In principle, a retrospective matched comparator cohort would be desirable; however, historical controls from the pre–complement inhibitor era would be subject to substantial temporal confounding and methodological challenges, thereby limiting the validity of direct comparisons. Moreover, we report a continuum of severe cases ranging from severe crisis‐near exacerbations where non‐invasive ventilation was sufficient and the ICU‐stay was relatively short. On the other end of the spectrum are cases with myasthenic crisis with prolonged invasive ventilation and lengthy ICU‐stays, with patients unresponsive to sequential standard and escalation therapies and with a higher number of complications. Furthermore, comparison between the eculizumab and the ravulizumab subgroup should be interpreted cautiously due to the low number of cases and important clinical differences between the two groups: patients receiving ravulizumab more frequently required non‐invasive ventilation, had shorter ICU stays and experienced fewer complications, whereas all six patients with prolonged ventilation (> 15 days) were in the eculizumab subgroup. As most eculizumab cases occurred during an earlier period with limited clinical experience using C5‐I in MG care, it is reasonable to assume that initiation of C5‐I therapy during myasthenic crisis may have been delayed. Likewise, one can speculate that the need to vaccinate patients may have contributed to delayed initiation of C5‐I therapy in some cases. This factor likely played a lesser role in later cases, when treating physicians had gained more experience with C5‐I and its associated vaccination requirements. We have therefore decided to report the results only descriptively. Moreover, in three cases, C5‐I therapy was initiated only one to three days before discharge from ICU. This implies that transferral from ICU was not dependent on the effect of C5‐I therapy—a therapy that can improve MG symptoms fast, but not within three days. However, we can only infer this conclusion due to the retrospective design of the study.

## 6 Outlook

Overall, the use of C5‐I in an impending myasthenic crisis might mark a shift in treatment strategies with earlier consideration of C5‐I as an option to stabilize a crisis‐like course of MG. Since mortality in myasthenic crisis remains high, the early use of fast‐acting targeted therapies like C5‐I might be an important tool to either prevent a myasthenic crisis altogether or shorten it and possibly also reduce the mortality. However, this needs to be proven in larger and prospective studies.

## 7 Conclusion

Emerging therapeutic strategies—including C5‐I (eculizumab, ravulizumab and zilucoplan)—offer new avenues for the management of (highly) active (refractory or unstable) gMG and may serve as bridge therapies in acute settings or as long‐term add‐on therapies. Yet, pivotal clinical trials have consistently excluded patients in myasthenic crisis, leaving the optimal timing and integration of these agents in crisis management unresolved. This gap in evidence underscores the need for focused investigation in high‐risk populations. Findings from our real‐world cohort suggest that add‐on complement inhibition with C5‐I in therapy‐refractory myasthenic crisis may shorten ICU or IMC stay and enable corticosteroid dose tapering, with clinical benefits observed even in patients with TAMG—a subgroup typically excluded from interventional studies. These observations together with the emerging evidence from the available literature provide a strong rationale to pursue prospective, systematically controlled multicentre studies. Such trials will be essential to formally establish the efficacy of C5‐I in myasthenic crisis and to define its role within future treatment algorithms.

## Author Contributions

**L.G.** wrote the manuscript. **L.G., A.M.** and **S.L.** developed the study design. **L.G., J.L., U.HvO., C.S., M.S., M.O.** conducted the study. All authors interpreted the data. Statistical analyses were performed by **A.S.** and **H.T.** the manuscript was critically reviewed and edited by all authors.

## Funding

The project was an Investigator‐initiated study led by the research team at the Charité—Universitätsmedizin Berlin. The research project was realized with financial support from Alexion Pharma Germany GmbH. Alexion was not involved in the design of the study or the analyses.

## Conflicts of Interest

L.G. received speaker's honoraria, honoraria for attendance at advisory boards and/or travel and congress fees from Alnylam, Alexion, Roche and UCB and is a shareholder of RareLink digital health GmbH. M.S. received speaker's honoraria and honoraria for attendance at advisory boards from Argenx, Alexion and UCB and is a shareholder of RareLink digital health GmbH. S.G. received speaker's honoraria for lecturing from Argenx, Alexion and UCB and travel, accommodation and congress fees from Grifols, UCB and Alexion. J.Z. received payments for advisory boards, speaker's honoraria, travel expenses, research projects from Alnylam, Biogen, Biotest, CSL Behring, Octapharma, Kedrion, Grifols, UCB, Hormosan, Alexion, and Sanofi. U. H.vO. reports speaker honoraria from Alexion and Hormosan. C.S. received honoraria for attendance at advisory boards, speaker's honoraria and/or travel expenses from Alexion, Argenx and Johnson&Johnson. C.H. received speaker honoraria, research support and travel expenses from BMS, Merck, Novartis, Roche. T.H. received research support from Biogen, Novartis GeneTherapies, Roche and Sanofi Genzyme, speakers and consultant honoraria from Biogen, Hormosan, Roche, Alexion, Novartis, Roche, Sanofi‐Genzyme, Alnylam and Argenx. M.O. received speaking honoraria and travel support from Amicus Therapeutics GmbH, Argenx Germany GmbH, CSL Behring GmbH, Janssen Cilag GmbH, and Novartis AG. She received travel support from ITF Pharma GmbH and Sanofi SA. She received research funding (paid to her institution) from the habilitation support program of the University Hospital Düsseldorf, the German Society for Muscle Diseases, the German GBS Foundation, and the EFRE/JTF program of the state of North Rhine Westphalia (EFRE‐20800340). T.R. reports grants from the German Ministry of Education, Science, Research and Technology, grants and personal fees from Sanofi‐Aventis and Alexion, personal fees from Biogen Idec, Roche, and Teva, and personal fees and nonfinancial support from Merck Serono. P.D. received speaker's honoraria from Argenx, Alexion and UCB, and honoraria for attendance at advisory boards from UCB. C.D. received speaker's honoraria from Alexion and UCB and received travel/accommodation expenses and honoraria for attendance at advisory board from Argenx. M.H. has received speaker's honoraria from Argenx and speaker's honoraria and honoraria for attendance at advisory boards from Alexion. S.H. has received received speaker's honoraria and honoraria for attendance at advisory boards from Alexion, Argenx, Roche, UCB and Grifols and research funding from Argenx and Janssen. P.M. received travel/accommodation/meeting expenses from UCB pharma. F.S. received travel/accommodation/meeting expenses from Alexion and Argenx and received speaking honoria and honoria for attendance at advisory boards from Alexion, Argenx and UCB and received research grants from Alexion Pharmaceuticals, argenx, Cytel and Octapharma. She serves as a member of the German myasthenia gravis society. A.M. received speaker or consultancy honoraria or financial research support (paid to his institution) from Alexion Pharmaceuticals, Argenx, Amgen, Axunio, Desitin, Grifols, Janssen, Merck, Novartis, Octapharma, Sanofi and UCB. He serves as member of the medical advisory board of the *German Myasthenia Gravis Society* and chairmen of the *Association for Research in Myasthenic Syndromes in Germany*. S.L. received travel/accommodation/meeting expenses from Alexion Astra Zeneca Rare Disease, Argenx, Johnson&Johnson, and UCB; she received speaking honoria and honoria for attendance at advisory boards from Alexion Astra Zeneca Rare Disease, Argenx, Biogen, Hormosan, Huma, Johnson&Johnson, Merck, Roche, StreamedUp and UCB. She received financial research support (paid to her institution) from Ad Scientiam, Alexion Pharmaceuticals, Argenx, Hormosan and UCB. S.L. is shareholder of RareLink digital health GmbH and mamahealth GmbH. Alice Schneider, Hanna Tangen, Johanna Loris, Melina Schlag, Julia Herzig‐Nichtweiß, Amani Suboh, and Lisa Schwarz have nothing to disclose.

## Supporting information


**Figure S1:** Flowchart of identified, excluded and included cases.
**Table S1:** Meningococcal Vaccination and antibiotic prophylaxis.
**Table S2:** Availability of Scores.

## Data Availability

The data that support the findings of this study are available from the corresponding author upon reasonable request.
