# Supplementary material for: Complement Inhibition Therapy in Myasthenic Crisis—A Multicentre Retrospective Analysis of 17 Cases From Germany
Source: Eur J Neurol. 2026 Apr 15;33(4):e70596. doi: 10.1111/ene.70596 (PMC13081792; doi:10.1111/ene.70596)
Supplement: Supplementary file 1 — Figure S1: Flowchart of identified, excluded and included cases. Table S1: Meningococcal Vaccination and antibiotic prophylaxis. Table S2: Availability of Scores. [file ENE-33-e70596-s001.pdf]

Supplement to:

Complement inhibition therapy in myasthenic crisis – a multicenter retrospective analysis of 17 cases from Germany

Authors:

Lea Gerischer[1,2], Maike Stein[1,2,3], Alice Schneider[3,4], Hanna Tangen[4], Johanna Loris[5], Stefanie Glaubitz[5], Jana Zschüntzsch[5], Ulrich Hofstadt-van Oy[6], Charlotte Schubert[7], Christoph Heesen[7], Melina Schlag[8], Tim Hagenacker[8], Menekse Oeztuerk[9,10], Tobias Ruck[9,10], Paolo Doksani[1,2], Carla Dusemund[1,2], Meret Herdick[1,2], Julia Herzig-Nichtweiß[1], Philipp Mergenthaler[1,11,12], Frauke Stascheit[1,2], Amani Suboh[1,2], Lisa Schwarz[1], Sarah Hoffmann[1,2], Andreas Meisel*[1,2,11], Sophie Lehnerer*[1,2,3]

[1] Department of Neurology with Experimental Neurology, Charité – Universitätsmedizin Berlin, corporate member of Freie Universität Berlin and Humboldt-Universität zu Berlin, Berlin, Germany
[2] Neuroscience Clinical Research Center, Charité – Universitätsmedizin Berlin, corporate member of Freie Universität Berlin and Humboldt-Universität zu Berlin, Berlin, Germany
[3] Berlin Institute of Health at Charité – Universitätsmedizin Berlin, Digital Health Center, Berlin, Germany
[4] Institute of Biometry and Clinical Epidemiology, Charité – Universitätsmedizin Berlin, corporate member of Freie Universität Berlin and Humboldt-Universität zu Berlin, Berlin, Germany
[5] Department of Neurology, University Medical Center Göttingen, Göttingen, Germany
[6] Department of Neurology, Knappschaft Kliniken Dortmund, Dortmund, Germany
[7] Institute of Neuroimmunology and MS (INIMS) and Department of Neurology, University Medical Center Hamburg-Eppendorf, Hamburg, Germany
[8] Department of Neurology and Center for Translational Neuro- and Behavioral Sciences (C-30 TBNS), University Medicine Essen, Essen, Germany
[9] Department of Neurology, Medical Faculty and University Hospital Düsseldorf, Heinrich Heine University Düsseldorf, Düsseldorf, Germany
[10] Department of Neurology with Heimer Institute for Muscle Research, University Hospital Bergmannsheil, Bochum, Germany
[11] Center for Stroke Research Berlin, Charité – Universitätsmedizin Berlin, Berlin, Germany
[12] Radcliffe Department of Medicine, University of Oxford, Oxford, UK
*AM and SL contributed equally

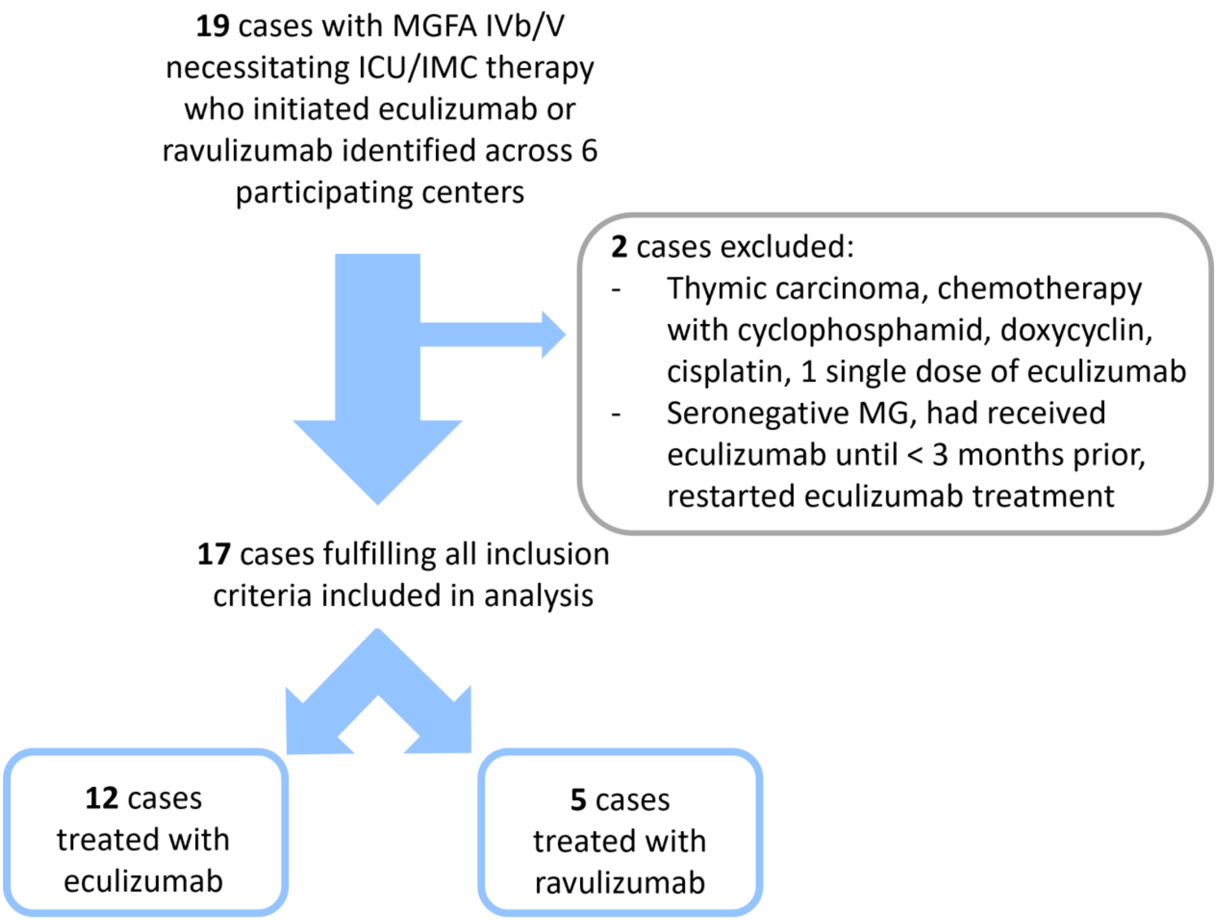

Supplementary Figure S1. Flowchart of identified, excluded and included cases.
The two excluded cases did not fulfil all inclusion criteria: one case was diagnosed with a thymic carcinoma and treated with chemotherapy and received only one single dose of eculizumab. We decided that a single dose did not qualify as "therapy start". The other case was a patient with seronegative MG, who had only interrupted therapy with eculizumab for less than three months and re-started eculizumab during the reported myasthenic crisis.
Abbreviations: ICU: intensive care unit; IMC: intermediate care unit; MG: myasthenia gravis; MGFA: Myasthenia Gravis Foundation of America Classification

Supplementary Table S1. Meningococcal Vaccination and antibiotic prophylaxis.

**Table S1. Meningococcal Vaccination and antibiotic prophylaxis**			
	**Overall N = 17**	**Eculizumab N = 12**	**Ravulizumab N = 5**
**Meningococcus ACWY + Meningococcus B vaccination**			
**Vaccination complete 2 weeks prior to ECU/RAVU**	4 (23.5%)	2 (16.7%)	2 (40%)
**Vaccination less than 2 weeks prior to ECU/RAVU**	12 (70.5%)	9 (75.0%)	3 (60.0%)
**Of those received antibiotic prophylaxis[a]**	11	9	2
**No information on antibiotic prophylaxis found**	1	0	1
**No vaccination data found[c]**	1 (5.8%)	1 (8.3%)	0
**Of those received antibiotic prophylaxis[b]**	1	1	0
[a]antibiotic prophylaxis if vaccination was done less than two weeks prior to ECU/RAVU:<br>3x ceftriaxone, 1x azithromycin, 1x Rifampicin, 2x Ampicillin, 2x Amoxicillin, 1x Penicillin G.<br>[b] antibiotic prophylaxis in case without vaccination prior to ECU/RAVU: Ciprofloxacin, then Penicillin V.<br>[c] the unvaccinated case belongs to the survivors.<br>Abbreviations: Ecu: eculizumab; Ravu: ravulizumab			

Supplementary Table S2. Availability of Scores

Table S2. Availability of Scores					
	**MGFA**	**MG-ADL**	**QMG**	**Besinger Score**	**Vital Capacity**
**Number of cases with score at least once**	17	10	13	7	11
**Number of measurements (total)**	99	46	50	40	57
**Measurements per patient, median [Min, Max]**	6.00 [3.00, 12.0]	3.50 [1.00, 11.0]	3.00 [1.00, 9.00]	7.00 [1.00, 8.00]	3.00 [1.00, 24.0]
Abbreviations: Max: maximum; Min: minimum; MG-ADL: Myasthenia Gravis Activities of Daily Living Score; MGFA: Myasthenia gravis Foundation of America Classification; QMG: Quantitative Myasthenia Gravis Score					
